# Rapid Resolution of Post-COVID-19 Inflammatory Syndrome in an Adult With Targeted Inhibition of Interleukin-1B

**DOI:** 10.7759/cureus.32078

**Published:** 2022-11-30

**Authors:** Mario Felix, Larabe Farrukh, Simple Modi, Ruben Peredo-Wende

**Affiliations:** 1 Department of Rheumatology, Yale School of Medicine, New Haven, USA; 2 Internal Medicine, Albany Medical Center, Albany, USA; 3 Department of Rheumatology, Albany Medical Center, Albany, USA; 4 Department of Rheumatology, Albany Stratton VA Medical Center, Albany, USA

**Keywords:** acute rheumatology, biologic agents, interleukin-1β inhibitor, multisystem inflammatory syndrome in adult, covid 19

## Abstract

Multisystem inflammatory syndrome (MIS) is a severe inflammatory response that occurs days to weeks following the infection of severe acute respiratory syndrome coronavirus 2 (SARS-CoV-2), the virus responsible for coronavirus disease 2019 (COVID-19). Initially known in children and named MIS-C, recently several cases of MIS in adults have been reported to the Centers for Disease Control and Prevention (CDC), leading to the recognition of a new disease MIS in adults (MIS-A). The current treatment options include high-dose steroids, intravenous immunoglobulin (IVIG), and immunosuppressive therapy. However, the pharmacologic approach remains limited to case reports and pending official guidelines to treat cases with MIS-A.

We present a case of an adult patient who had a severe inflammatory state following COVID-19 infection, who was treated with IL-1 antagonist therapy with a successful outcome.

## Introduction

Multisystem inflammatory syndrome (MIS) is a severe inflammatory response, days to weeks following the infection of severe acute respiratory syndrome coronavirus 2 (SARS-CoV-2), the virus responsible for coronavirus disease 2019 (COVID-19) infection. It may be associated with a systemic inflammatory response affecting multiple organs resulting in shock, cardiac dysfunction, abdominal pain, and elevated inflammatory markers including erythrocyte sedimentation rate (ESR), C-reactive protein (CRP), ferritin, D-dimers, and interleukin-6 (IL-6). Initially known in children and named MIS-C, starting June 2020 several cases of MIS in adults have been reported to the Centers for Disease Control and Prevention (CDC), leading to the recognition of a new disease MIS in adults (MIS-A). MIS should be carefully differentiated from a biphasic acute COVID-19 infection or a continuation of an unresolved COVID-19 inflammatory state. The treatment options include high-dose glucocorticoids, intravenous immunoglobulin (IVIG), and immunosuppressive therapy. However, the pharmacologic therapy approach is still limited to case reports and pending official guidelines to treat cases with MIS-A [1,2].

We present a case of an adult patient who developed MIS-A following COVID-19 infection, who was managed based on interleukin-1 antagonist therapy. The outcome was successful after several days of treatment and he was discharged under stable conditions.

## Case presentation

We present a 38-year-old male with a history of asthma, type-2 diabetes mellitus, and obesity who presented to the hospital with worsening symptoms after previously receiving inpatient treatment for COVID-19 pneumonia.

Seventeen days before admission day, he tested COVID-19 positive with a nasopharyngeal swab for polymerase chain reaction (PCR) SARS-CoV-2. He was asymptomatic at the time and was sent home for quarantine. Ten days before admission, he developed subjective fever and chills, dyspnea, productive cough, and diffuse myalgia. On presentation, chest radiograph showed bilateral peribronchial opacities consistent with pneumonia. EKG showed sinus tachycardia with non-specific T-wave changes. Labs showed leukocytosis of 11,800/uL (4000-9000/uL), CRP of 81.2 mg/L (<8.0 mg/L), ferritin of 1987 ng/mL (24 - 336 ng/mL), and a troponin of 0.07 ng/mL (0.00-0.04 ng/mL). He was started on a five-day course of 6 mg daily dexamethasone, along with azithromycin for COVID-19 pneumonia. The patient was discharged after clinical and laboratory improvement (CRP 61.6 mg/L) and treatment completion.

One day after discharge, his dyspnea re-emerged with symptoms at rest. He endorsed continuous high fever (Tmax 106 F), disabling myalgia, muscular weakness, upper extremity numbness and swelling, and polyarthralgia. On presentation to the hospital, his heart rate was elevated to 110 beats/min, respiratory rate was 45 breaths/min. He was started on 2 Liters/min O2 via nasal cannula. Physical exam revealed rales and wheeze in bilateral lower lung fields. SARS-CoV-2 PCR was still positive. Chest CT-angiography excluded embolism, showing residual peri-bronchial opacities. Laboratories were: WBC 21.200/uL (4000-9000/uL), CRP 187.4 mg/L (<8.0 mg/dL), ESR 49 mm/hr (0-15 mm/hr), ferritin 2842 ng/mL (24-336 ng/mL), D-dimer 0.79 mg/L (<0.5 mg/dL), fibrinogen 789 mg/dL (172-483 mg/dL), troponin 0.05 (0.00-0.04 ng/mL).

CT abdomen/pelvis did not show any infectious foci. Due to complaints of hoarseness of voice and globus sensation, CT soft tissue of the neck was ordered which did not reveal any abnormalities. MRI of the cervical spine was ordered due to complaints of upper extremity numbness and weakness which only showed edema in his dorsal paraspinal muscles and cervical ligaments. Broad-spectrum antibiotics were administered in the form of meropenem and linezolid. Repeat cultures obtained during this time were negative. By day 12, the patient showed no signs of improvement. Repeat serum laboratories showed an overall worsening of his inflammatory markers; especially his ferritin level. Echocardiogram obtained to look for evidence of endocarditis was unremarkable. A tagged WBC scan did not show any hidden infectious foci. 

The rheumatology service was consulted and raised concern for post-COVID-19 MIS-A. He was started on 100 mg anakinra twice a day. Within two to three days of starting anakinra, the patient showed a marked improvement in his clinical symptoms co-relating to an almost 50% decrease in his inflammatory markers. His mental status and respiratory status improved, strength returned, and myalgias resolved. Antibiotics were discontinued and the patient continued on anakinra for the rest of the hospital course. He continued to improve clinically and his inflammatory markers decreased. He was discharged from the hospital on day 27 (Table 1). 

**Table 1:** Laboratory findings in the presented case WBC: white blood cell, CRP: C-reactive protein, ESR: erythrocyte sedimentation rate, PCR: polymerase chain reaction, SARS-CoV-2: severe acute respiratory syndrome coronavirus 2, IgG: immunoglobulin G

Labs (normal range)	Day 12 of admission	Day 7 of Anakinra therapy
WBC count (4000-9000/uL)	25,000/uL	6,300/uL
CRP (<8.0 mg/dL)	187.4 mg/L	6.8 mg/L
ESR (0-15 mm/hr)	122 mm/hr	87 mg/dL
Ferritin (24 - 336 ng/mL)	12740ng/mL	1772 ng/mL
D-dimer (<0.5 mg/dL)	15.17 mg/L	6.19 mg/dL
Fibrinogen (172-483 mg/dL)	789 mg/dL	428 mg/dL
Pro-calcitonin (<0.5 ng/ml)	6.2 ng/ml	0.82 ng/ml
SARS CoV-2 PCR and IgG	Positive	-

The plan was to continue anakinra 100 mg subcutaneously after discharge and follow up with Rheumatology as an outpatient. He continued to take anakinra daily and had an improvement in ferritin levels. The patient complained of having persistent bilateral lower extremity pain and weakness, for which he was prescribed a tapering course of prednisone, which resulted in improvement. 

## Discussion

MIS-A is a rare complication of SARS-CoV-2 infection. It is believed to result from a dysregulated immune response to the virus [3]. Despite several cases reported in the literature, MIS-A lacks an accepted definition so far. The case definition made by the CDC requires five criteria to be present: (1) a severe illness requiring hospitalization in a person aged ≥21 years; (2) a positive test result for current or previous SARS-CoV-2 infection in the last 12 weeks; (3) severe dysfunction of one or more extra-pulmonary organ systems; (4) laboratory evidence of severe inflammation; (5) absence of severe respiratory illness (to exclude patients in which inflammation and organ dysfunction might be attributable simply to tissue hypoxia); alternative diagnoses must be excluded (Table 2) [4].

**Table 2:** Centers for Disease Control and Prevention Criteria for MIS-A MIS-A: Multisystem Inflammatory Syndrome in Adults, LVEF: left ventricular ejection fraction, SARS-CoV-2: severe acute respiratory syndrome coronavirus 2, RT-PCR: reverse transcription polymerase chain reaction

Case Definition of MIS-A	Present in this case
Patient aged ≥21 years hospitalized for ≥24 hours	Yes
Exclusion of alternative diagnosis (e.g., bacterial sepsis, exacerbation of a chronic medical condition)	Yes
Fever (≥38.0 C) for ≥24 hours prior to hospitalization or within the first THREE days of hospitalization	Yes
Clinical Criteria	
Primary clinical criteria	
Severe cardiac illness (myocarditis, pericarditis, coronary artery dilatation/aneurysm, or new-onset right or left ventricular dysfunction (LVEF<50%), 2nd/3rd degree A-V block, or ventricular tachycardia)	No
Rash and non-purulent conjunctivitis	No
Secondary clinical criteria	
New-onset neurologic signs and symptoms (encephalopathy, seizures, meningeal signs, or peripheral neuropathy)	Yes
Shock or hypotension not attributable to medical therapy	No
Abdominal pain, vomiting, or diarrhea	No
Thrombocytopenia (platelet count <150,000/ microliter)	No
Laboratory evidence	
Elevated levels of at least TWO of the following: C-reactive protein, ferritin, IL-6, erythrocyte sedimentation rate, procalcitonin	Yes
A positive SARS-CoV-2 test for current or recent infection by RT-PCR, serology, or antigen detection	Yes

Our patient had a SARS-CoV-2 positive PCR as well as positive IgG antibodies, with significant lab evidence of an inflammatory disorder. Infectious work-up including blood cultures, respiratory cultures, and urine cultures were all negative. Chest X-ray and CT scan showed ground-glass attenuation and peri-bronchial consolidation in bilateral lower lobes, which were most likely secondary to previous SARS-CoV-2 infection. Superimposed pneumonia infection was considered; however, the patient did not have any improvement in symptoms despite receiving broad-spectrum antibiotics. The lack of mucocutaneous involvement, and lymphadenopathy excluded Kawasaki disease. Drug reaction with eosinophilia and systemic symptoms post antibiotic use was excluded as eosinophils were within normal limits. Other systemic inflammatory conditions like hemophagocytic lymphohistiocytosis (HLH) were specifically ruled out due to the absence of any cytopenia, splenomegaly, hypertriglyceridemia, or hypofibrinogenemia. Due to the constellation of symptoms, recent SARS-CoV-2 infection, and rapid clinical deterioration despite broad-spectrum antibiotics, a differential diagnosis of MIS-A related to COVID-19 was considered. 

Multi-system inflammatory syndrome was initially described as a Kawasaki-like or toxic-shock syndrome-like disease that occurred primarily in children after acute COVID-19 infection. Although thought to be a rare finding in adults, there has been a multitude of recently published case reports that describe this syndrome and its variety of presentations in adults [5]. We report here an adult male with post-COVID-19 inflammatory syndrome, who displayed robust clinical improvement with an interleukin-1B (IL-1B) inhibitor, anakinra monotherapy.

IL-1B has been identified as one of the key cytokines mediating the hyper-inflammatory state associated with COVID-19 infection. Anakinra is a recombinant IL-1B receptor antagonist that has been used as a pharmacotherapeutic option in children with MIS-C, although there is a lack of data publishing its efficacy in adults. There are a few published case reports of young males with multi-system inflammatory syndrome who improved with combination therapy consisting of pulse-dose steroids, anakinra, and IVIG therapy [6,7].

## Conclusions

Our report describes an important case that demonstrates rapid improvement of post-COVID-19 inflammatory syndrome with anakinra monotherapy in an adult patient. This report serves as evidence of another option to treat post-COVID-19 inflammation in adults, especially in patients who may have a contraindication or could be intolerant to steroid therapy, such as our patient who had uncontrolled diabetes mellitus.

## References

[1] Feldstein LR, Rose EB, Horwitz SM (2020). Multisystem inflammatory syndrome in US children and adolescents. N Engl J Med.

[2] Brown LM, Semler MW, Hansen M, Person AK, Kelly SG (2021). Multisystem inflammatory syndrome in an adult with COVID-19. Infect Dis Clin Pract (Baltim Md).

[3] Maltezou HC, Pavli A, Tsakris A (2021). Post-COVID syndrome: an insight on its pathogenesis. Vaccines (Basel).

[4] (2022). Multisystem inflammatory syndrome in adults (MIS-A): case definition. https://www.cdc.gov/mis/mis-a/hcp.html#:~:text=Case%20Definition%20for%20MIS%2DA&text=Subjective%20fever%20or%20documented%20fever,first%20THREE%20days%20of%20hospitalization*..

[5] Cogan E, Foulon P, Cappeliez O, Dolle N, Vanfraechem G, De Backer D (2020). Multisystem inflammatory syndrome with complete Kawasaki disease features associated with SARS-CoV-2 infection in a young adult. A case report. Front Med (Lausanne).

[6] Cattaneo P, Volpe A, Cardellino CS (2021). Multisystem inflammatory syndrome in an adult (MIS-A) successfully treated with anakinra and glucocorticoids. Microorganisms.

[7] Aggarwal A, Cohen E, Figueira M (2021). Multisystem inflammatory syndrome in an adult with COVID-19-a trial of anakinra: a case report. Infect Dis Clin Pract (Baltim Md).

